# Activation of peripheral KCNQ channels attenuates inflammatory pain

**DOI:** 10.1186/1744-8069-10-15

**Published:** 2014-02-21

**Authors:** Hiroki Hayashi, Masashi Iwata, Noboru Tsuchimori, Tatsumi Matsumoto

**Affiliations:** 1 Pharmaceutical Research Division, Inflammation Drug Discovery Unit, Takeda Pharmaceutical Company Limited, Fujisawa, Kanagawa, Japan

**Keywords:** KCNQ, Retigabine, ICA-27243, XE-991, Inflammatory pain

## Abstract

**Background:**

Refractory chronic pain dramatically reduces the quality of life of patients. Existing drugs cannot fully achieve effective chronic pain control because of their lower efficacy and/or accompanying side effects. Voltage-gated potassium channels (KCNQ) openers have demonstrated their analgesic effect in preclinical and clinical studies, and are thus considered to be a potential therapeutic target as analgesics. However, these drugs exhibit a narrow therapeutic window due to their imposed central nerve system (CNS) side effects. To clarify the analgesic effect by peripheral KCNQ channel activation, we investigated whether the analgesic effect of the KCNQ channel opener, retigabine, is inhibited by intracerebroventricular (i.c.v.) administration of the KCNQ channel blocker, 10, 10-bis (4-Pyridinylmethyl)-9(10H) -anthracenone dihydrochloride (XE-991) in rats.

**Results:**

Oral administration (p.o.) of retigabine showed an anticonvulsant effect on maximal electronic seizures and an analgesic effect on complete Freund’s adjuvant-induced thermal hyperalgesia. However, impaired motor coordination and reduced exploratory behavior were also observed at the analgesic doses of retigabine. Administration (i.c.v.) of XE-991 reversed the retigabine-induced anticonvulsant effect, impaired motor coordination and reduced exploratory behavior but not the analgesic effect. Moreover, intraplantar administration of retigabine or an additional KCNQ channel opener, N-(6-Chloro-pyridin-3-yl)-3,4-difluoro-benzamide (ICA-27243), inhibited formalin-induced nociceptive behavior.

**Conclusions:**

Our findings suggest that the peripheral sensory neuron is the main target for KCNQ channel openers to induce analgesia. Therefore, peripheral KCNQ channel openers that do not penetrate the CNS may be suitable analgesic drugs as they would prevent CNS side effects.

## Background

Chronic pain is resistant to analgesics, and dramatically reduces the quality of life of patients. Non-steroidal anti-inflammatory drugs and opioids are analgesics used for acute pain, and also anticonvulsants and antidepressants are used as supplementary analgesics for convulsions and depression associated with chronic pain [1-4]. However, all these drugs cannot fully achieve effective chronic pain control because of their lower efficacy and/or accompanying side effects [1-4]. Therefore, development of novel analgesics for chronic pain is highly anticipated.

Flupirtine (Katadolon®) is a centrally acting non-opioid analgesic (available in Germany) for the treatment of a variety of pain states, including chronic pain, such as low back pain and cancer pain [5-10]. However, flupirtine is not universally used because of its side effects on the central nervous system (CNS), such as somnolence, dizziness, and nausea [5-10]. Although the mechanism of action of flupirtine has not yet been fully elucidated, it was recently reported to inhibit neural excitability via the opening of voltage-gated potassium channels (KCNQ) [11,12]. Therefore, the KCNQ channel may be a potential drug target for analgesics; however, CNS side effects may accompany its therapeutic effect.

The KCNQ channel consists of four KCNQ subunits as a homo- or hetero-tetramer [13]. There are five KCNQ genes coding for five KCNQ subunits, KCNQ1-5 [12]. KCNQ2-5 are expressed in the CNS and peripheral nervous system (PNS), such as primary afferents [14-19]. Neuronal M-currents are carried via a heteromultimeric combination of KCNQ2, KCNQ3, and KCNQ5 [20,21], and are activated at potentials that are at a sub-threshold for action potential firing and control resting membrane potential [14,20]. Therefore, KCNQ channels play an important role in regulating neuronal excitability. The relationship of KCNQ channels with the control of sensory nerve excitability and pain processing is demonstrated by preclinical pharmacological studies using retigabine, the analogue of flupirtine, which has higher KCNQ channel opening activity than flupirtine [22-25]. Electrophysiological studies using isolated spinal cord or small diameter dorsal root ganglion cells have shown that retigabine raises the threshold of firing by hyperpolarizing the membrane potential [23,26,27]. Moreover, retigabine inhibits spinal dorsal horn neuronal firing rate induced by electrical stimulation in both naïve and spinal nerve ligated rats in vivo [23]. In behavioral studies, retigabine has been reported to exert analgesic effects in noxious, inflammatory, and neuropathic pain models [23,28,29]. However, retigabine has displayed CNS side effects, such as impaired motor coordination and reduced exploratory behavior at similar doses to those showing an analgesic effect [30-32].

The KCNQ channel opener, retigabine, has been approved as a therapeutic drug for refractory partial-onset seizures [33,34]. In clinical trials, dose-limiting CNS side effects associated with retigabine, such as dizziness, somnolence and fatigue were observed at therapeutic doses [30,35]. An additional KCNQ channel opener, *N*-(6-chloro-pyridin-3-yl)-3,4-difluoro-benzamide (ICA-27243), is structurally different from retigabine [36,37]. ICA-27243 exhibits higher selectivity than retigabine as a KCNQ2/3 channel opener, and also occupies a narrow therapeutic window against convulsions and CNS side effects [37]. These findings suggest that KCNQ channel opening activity results in CNS side effects, which may not be prevented by only selectively activating KCNQ2/3. In contrast, KCNQ channel openers that do not penetrate the CNS are assumed to have no CNS side effects. However, because the site of action for their analgesic effect is not fully elucidated, the dissociation between this effect and CNS side effects remains to be resolved.

In the present study, we aimed to clarify the site of action for the analgesic effect of KCNQ openers by investigating the contribution of brain KCNQ channel opening activity with reduced exploratory behavior, impaired motor coordination, and analgesic effects.

## Results

### Analgesic effect of retigabine and ICA-27243 in CFA-induced thermal hyperalgesia

**Figure 1:**
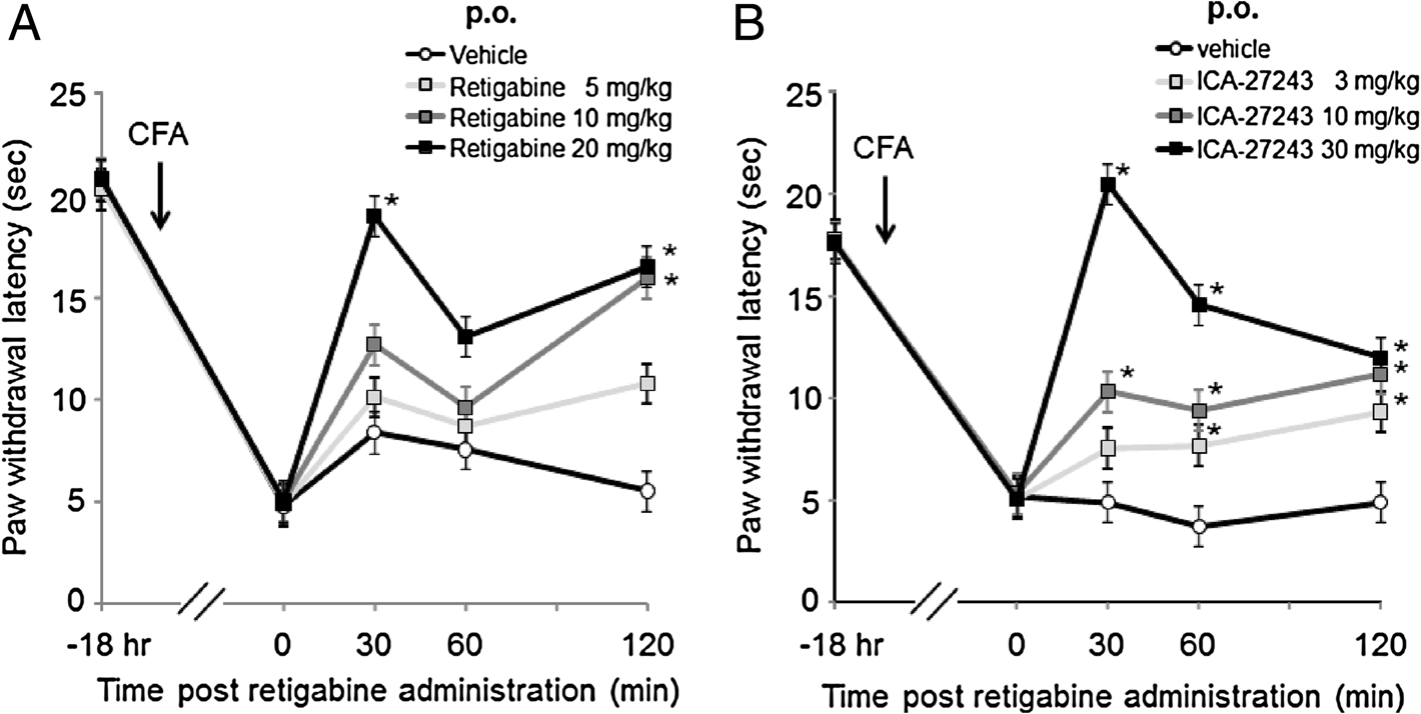
**Analgesic effect of retigabine and ICA-27243 in complete Freund’s adjuvant-induced inflammatory pain model.** Rats exposed to retigabine **(A)** and ICA-27243 **(B)** at 5, 10 or 20 mg/kg and 3, 10 or 30 mg/kg were tested for PWL 30, 60 and 120 min after the dosing. Each data was presented as the mean ± S.E.M. of determinations in 6 rats. *p < 0.05 vs vehicle.

Analgesic effects of retigabine (5–20 mg/kg) and ICA-27243 (3–30 mg/kg) in Complete Freund’s adjuvant (CFA) -induced thermal hyperalgesia were evaluated by the plantar test at 30, 60 and 120 min post administration (Figure 1A, B). Orally administered (p.o.) retigabine or ICA-27243 dose-dependently increased paw withdrawal latency (PWL) and significantly effects were observed 30 min after administration of 20 mg/kg retigabine or 10 and 30 mg/kg ICA-27243 (*p* < 0.05).

### Retigabine and ICA-27243 impaired motor coordination

**Figure 2:**
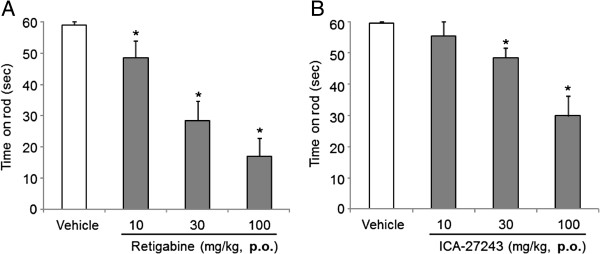
**Retigabine or ICA-27243 impairs motor coordination.** Rats administered with retigabine **(A)** and ICA-27243 **(B)** at 10, 30 or 100 mg/kg were tested for time spent on the rotarod 30 min after the dosing. Each data was presented as the mean ± S.E.M. of determinations in 6 rats. *p < 0.05 vs vehicle.

Vehicle-treated rats spent approximately 60 sec (cut-off time) on the rod (Figure 2A, B). Compared with vehicle, orally administered retigabine or ICA-27243 (both at 10, 30, or 100 mg/kg) reduced the running latency on the rod in a dose-dependent manner. Compared with vehicle, a significantly reduction was observed with 10, 30 or 100 mg/kg retigabine and 30 or 100 mg/kg ICA-27243 (*p* < 0.05).

### Retigabine and ICA-27243 decreased exploratory behavior

**Figure 3:**
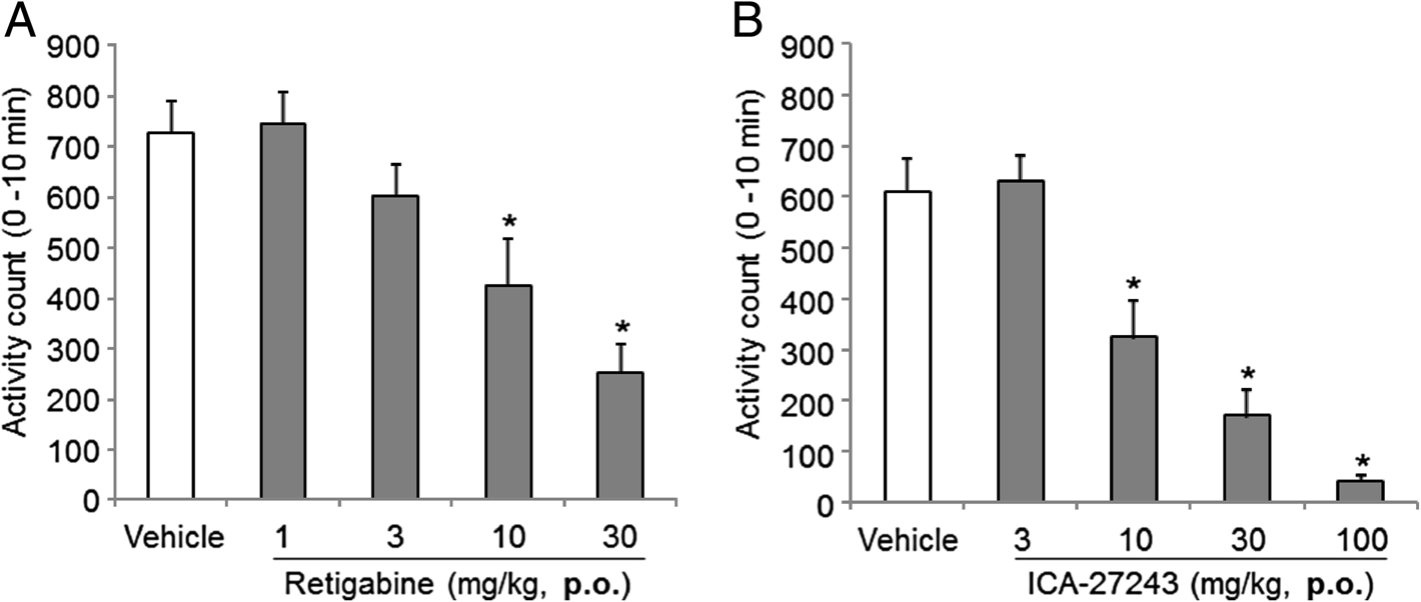
**Retigabine or ICA-27243 reduces exploratory behavior.** Exploratory behavior in rats was quantified 30 min after the treatment with retigabine **(A)** and ICA-27243 **(B)** at 1, 3, 10 or 30 mg/kg and 3, 10, 30 or 100 mg/kg, respectively. Each data was presented as the mean ± S.E.M., each mean showed summation for 10 min in 6 rats. *p < 0.05 vs vehicle.

Activity counts of exploratory behavior of rats treated with vehicle prior to retigabine or ICA-27243 administration were 727 ± 63 or 611 ± 66, respectively (Figure 3A, B). Compared with vehicle, orally administered retigabine or ICA-27243 dose-dependently decreased exploratory behavior and significantly effects were observed with 10 or 30 mg/kg retigabine and 10, 30 or 100 mg/kg ICA-27243 (*p* < 0.05).

### XE-991 reversed retigabine-induced anticonvulsant activity

**Figure 4:**
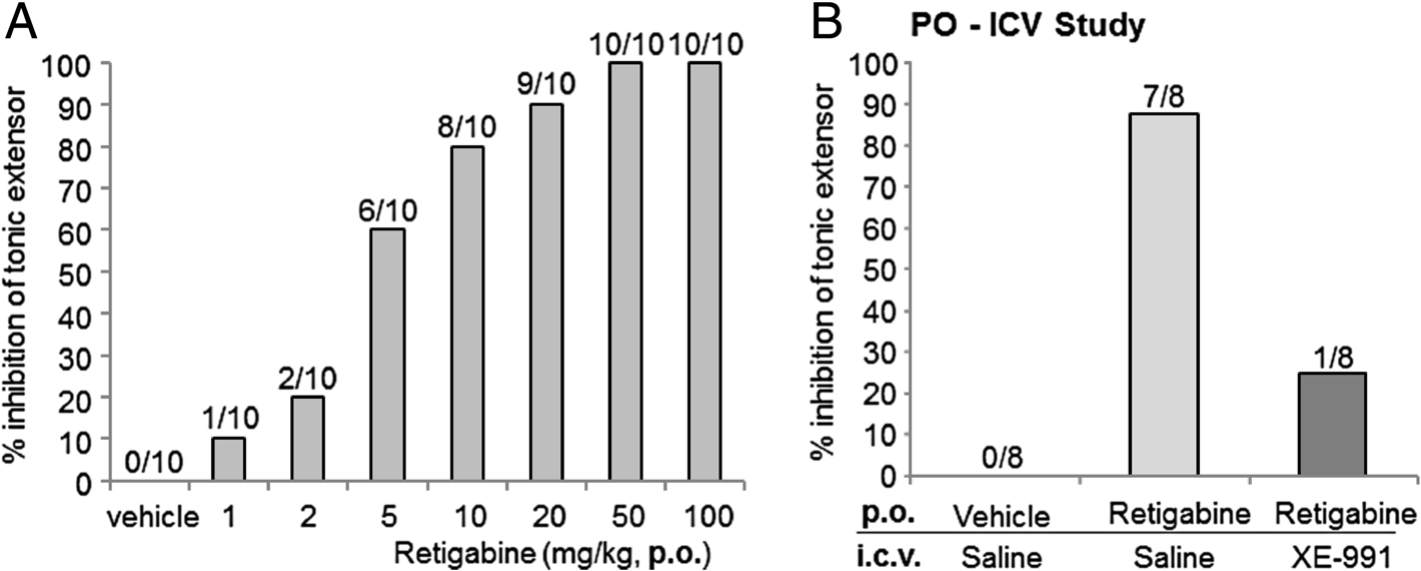
**XE-991 reverses retigabine-induced anticonvulsant activity. (A)** The maximal electronic seizures (MES) test was used to determine tonic extensor convulsions in rats exposed to retigabine at 1–100 mg/kg. **(B)** Retigabine (20 mg/kg)-exposed rats with or without XE-991 (80 μg/site) were tested for anticonvulsant activity via the MES test. Retigabine and XE-991 was administered 30 min and 20 min prior to the MES test, respectively. Numbers on top of each column represent the number of rats in which tonic extensor convulsions were inhibited out of total number (N = 10 and 8, respectively).

Using the maximal electroshock seizure (MES) test, rats treated with vehicle were shown to develop tonic convulsions (Figure 4A), and the administration of retigabine dose-dependently reduced these electroshock-induced convulsions. Retigabine inhibited tonic convulsions by approximately 90%. After intracerebroventricular (i.c.v.) injection of 80 μg XE-991 20 min beforehand, this inhibition was reduced to 25% (Figure 4B). The dose of 80 μg/site of XE-991 was used in the following behavioral tests because convulsion-like behaviors were sometimes observed after i.c.v. injection of XE-991 at doses equal to or exceeding 100 μg/site (data not shown).

### XE-991 reversed retigabine-induced motor coordination impairment

**Figure 5:**
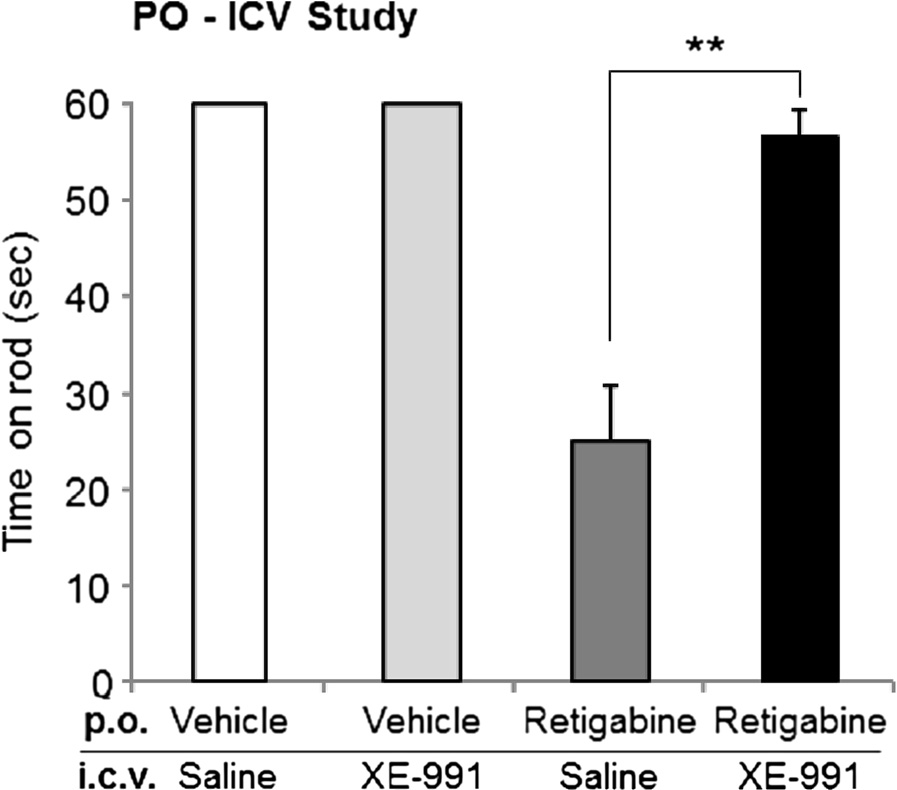
**XE-991 reverses retigabine-induced motor coordination impairment.** Motor coordination impairment was quantified in retigabine (50 mg/kg)-exposed rats with or without XE-991 (80 μg/site). Retigabine and XE-991 was administered 30 min and 20 min prior to the behavior test, respectively. Each data was presented as the mean ± S.E.M. in 10 rats. **p < 0.01.

We next investigated the effect of XE-991 on retigabine-induced motor coordination impairment via the rotarod test. Intracerebroventricular injection of saline or XE-991 alone did not affect the latency to fall (mean time on the rod in both groups = 60 sec [cut-off time], data not shown). Retigabine reduced the time on rod to 25.1 ± 5.7 sec. However, after i.c.v. injection of 80 μg XE-991 20 min beforehand, this reduction was reversed to 56.5 ± 3.1 sec (Figure 5). These results suggested that opening of brain KCNQ channels may be responsible for retigabine-induced motor coordination impairment.

### XE-991 reversed retigabine-induced reduction of exploratory behavior

**Figure 6:**
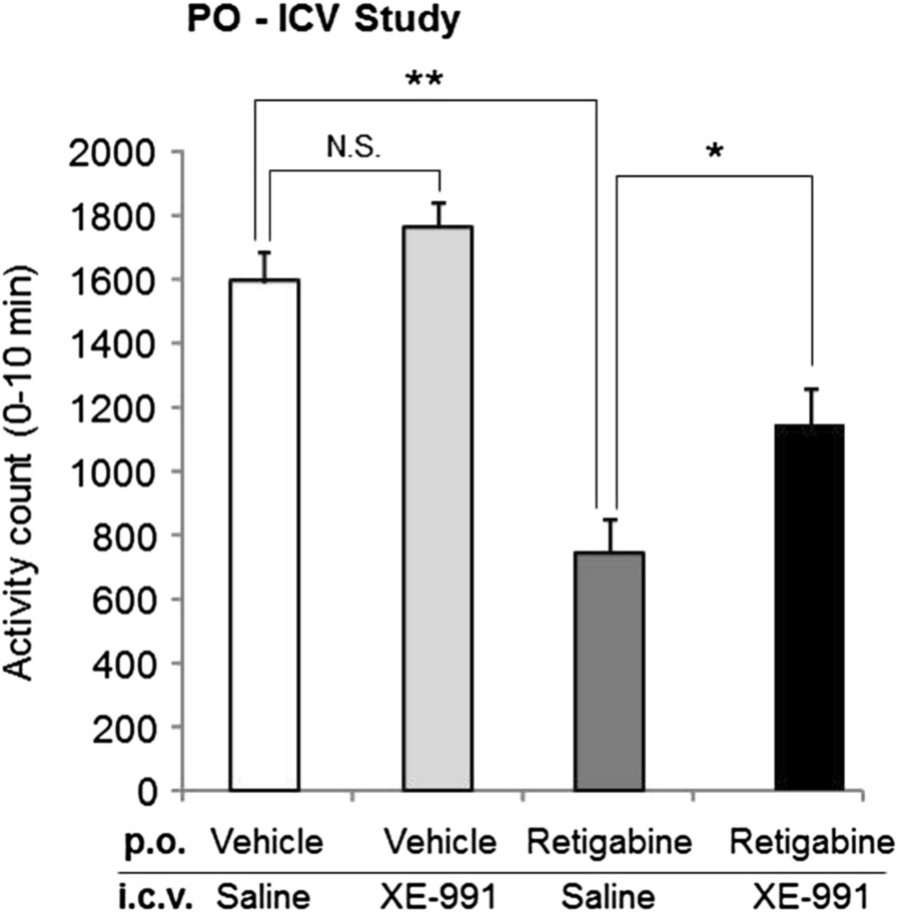
**XE-991 reverses retigabine-induced reduction in exploratory behavior.** Exploratory behavior was assessed in rats exposed to retigabine (20 mg/kg) with or without XE-991 (80 μg/site). Retigabine and XE-991 was administered 30 min and 20 min prior to the behavior test, respectively. Each data was presented as the mean ± S.E.M. from the 0 to 10 min assessment period (i.e., 30 to 40 min post-oral administration) in 27 rats. Data were pooled from 3 independent experiments. *p < 0.05, **p < 0.01; N.S., not significant.

XE-991 was then investigated for its effect on retigabine-mediated reduction of locomotor activity. Intracerebroventricular injection of 80 μg XE-991 alone did not affect locomotor activity (data not shown). Activity counts of vehicle-treated rats with saline or XE-991 were 1593 ± 95 or 1762 ± 83, respectively (Figure 6) and the difference between the two groups was not significant. Retigabine significantly reduced locomotor counts to 746 ± 101 (*p* < 0.01). After i.c.v. injection of 80 μg XE-991 20 min beforehand, this decrease in number was (yet partially) reversed to 1142 ± 116 (*p* < 0.05) (Figure 6). These results indicated that activation of brain KCNQ channels was partially involved in retigabine-induced reduction of exploratory behavior.

### XE-991 did not affect retigabine-induced analgesia in an inflammatory pain model

**Figure 7:**
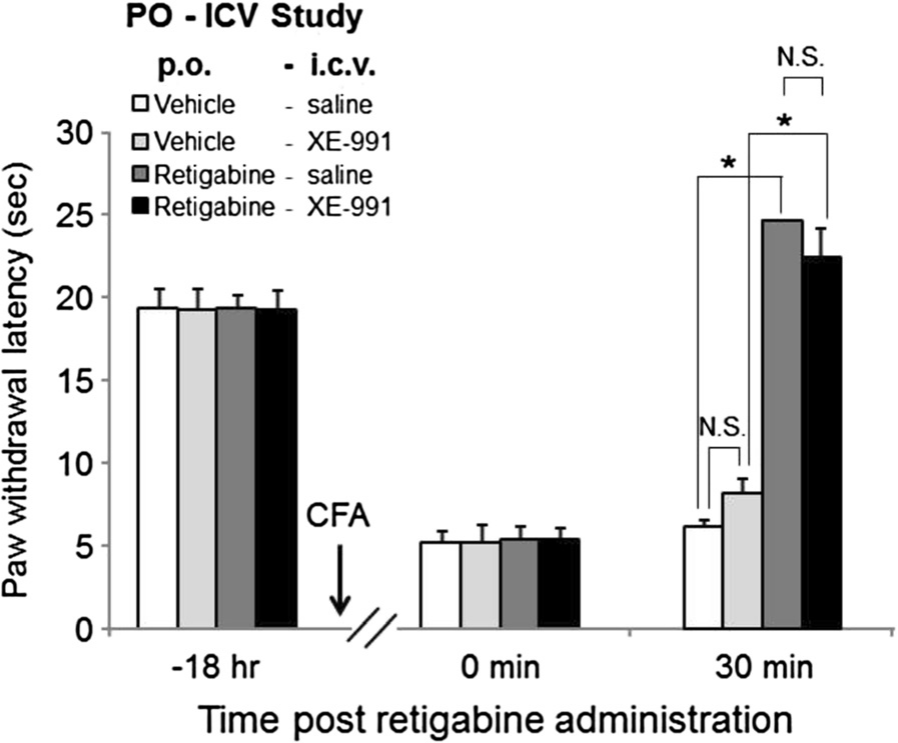
**XE-991 reversed retigabine-induced analgesia in the inflammatory pain model.** Inflammatory pain was induced by CFA injection, and 18 hr after CFA injection, PWL was measured in retigabine (20 mg/kg)-exposed rats with or without XE-991 (80 μg/site). Retigabine and XE-991 was administered 30 min and 20 min prior to the behavior test, respectively. Each data was presented as the mean ± S.E.M. in 5 to 6 rats. *p < 0.05; N.S., not significant.

To determine if KCNQ channel openers produce an analgesic effect via the brain, XE-991 was examined for its effect on retigabine-mediated reversal of CFA-induced thermal hyperalgesia. The PWL of vehicle-treated rats with XE-991 was 8.1 ± 0.9 sec while vehicle-treated rats with saline was 6.2 ± 0.4 sec, and the difference between the two groups was not significant. Thus XE-991 did not affect the hyperalgesia (Figure 7). Retigabine-treated rats exhibited an increase in PWL to 24.7 ± 0.2 sec; however, after i.c.v. injection of 80 μg XE-991 20 min beforehand, this effect was not significantly changed (22.4 ± 1.8 sec) (Figure 7). These results suggested that brain KCNQ channels may not be involved in retigabine-mediated reversal of CFA-induced thermal hyperalgesia.

### Intraplantar injection of retigabine or ICA-27243 induced an analgesic effect

**Figure 8:**
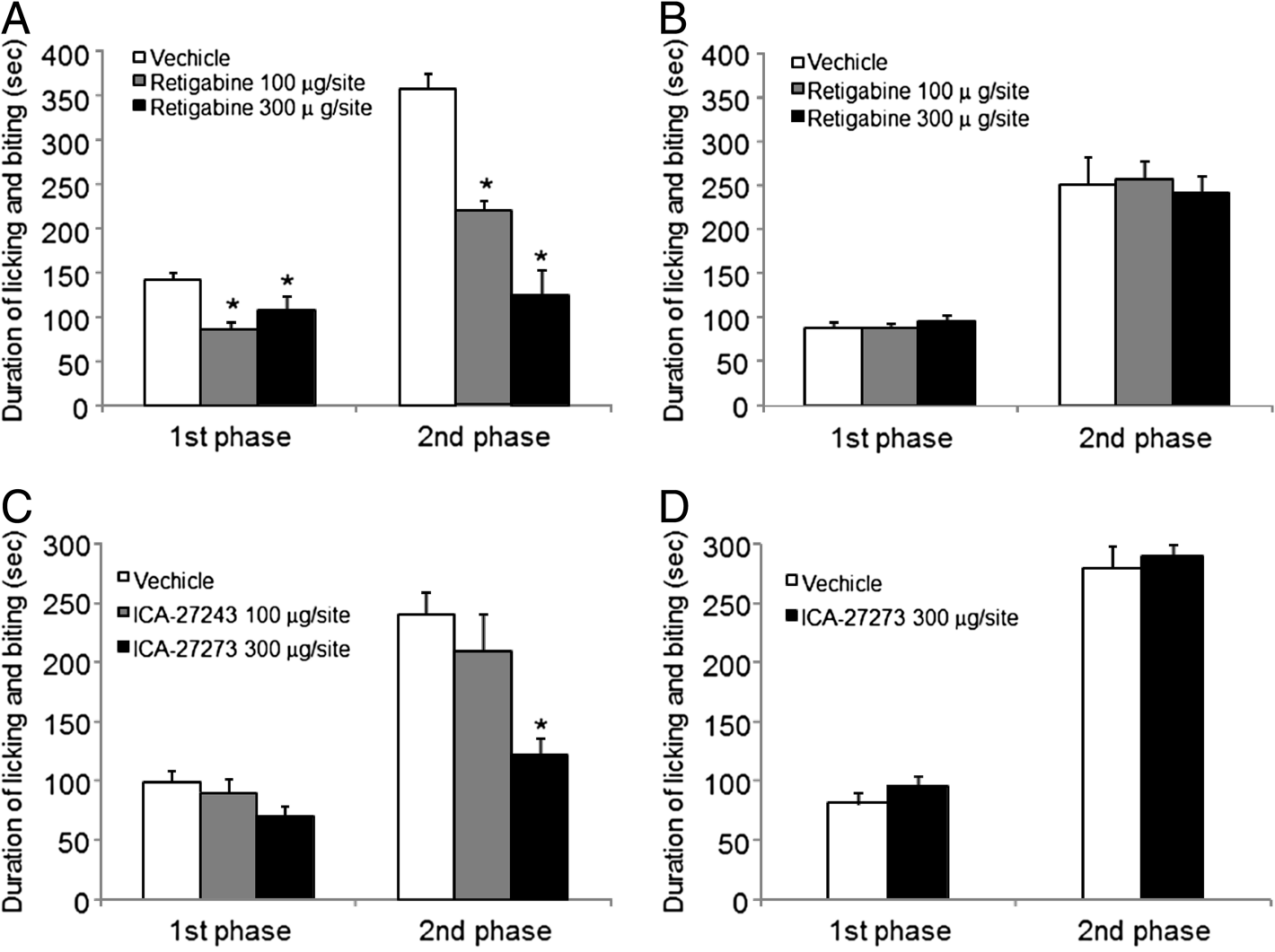
**Analgesic effect of intraplantar administration of retigabine and ICA-27243.** Nociceptive behavior was induced by an intraplantar injection of formalin. Ten min prior to the formalin injection, 100 and 300 μg retigabine and ICA-27243 was injected into intraplantar surface of the ipsilateral paw **(A & C)**, respectively and of the contralateral paw **(B & D)**, respectively. The duration of licking behavior was recorded 0–10 min (1st phase) and 10–30 min post-formalin injection (2nd phase). Each data was presented as the mean ± S.E.M. in 6 rats. *p < 0.05.

Some reports showed the intraplantar injection of retigabine attenuated bradykinin induced nociceptive behavior, suggesting that retigabine produces an analgesic effect via peripheral nerves [38,39]. To confirm the suggestion, the effect of local injection of retigabine or ICA-27243 was tested in formalin-induced nociceptive behavior. Retigabine or ICA-27243 significantly inhibited formalin-induced licking behavior during the first (0–5 min) and second (10–30 min) phases (Figure 8). Compared with vehicle, licking time in the first phase was significantly reduced by 100 or 300 μg/site retigabine by 40% or 25%, respectively (*p* < 0.05). Moreover, licking time in the second phase was significantly reduced by 100 or 300 μg/site retigabine by 38% or 65%, respectively (*p* < 0.05) (Figure 8A). Similar significantly results were found with ICA-27243 (*p* < 0.05) (Figure 8C): 9% or 29% with 100 or 300 μg/site ICA-27243, respectively in the first phase, and 13% or 50% with 100 or 300 μg/site ICA-27243, respectively in the second phase. However, administration of retigabine or ICA-27243 (300 μg/site) on the contralateral side did not affect formalin-induced licking time (Figure 8B and D).

## Discussion

Noxious stimuli generate action potentials at peripheral nerve endings of nociceptive neurons. Impulses that pass through primary sensory nerves into the spinal cord dorsal horn are brought to the cerebral cortex via the spinothalamic tract, thus conveying pain signals [40,41]. Therefore, blocking the action potentials at any region in this signaling tract would be expected to result in analgesia. KCNQ channels (except KCNQ1) have been shown to be expressed in the primary afferent, spinal cord and brain [14-19]. Retigabine was reported to inhibit discharges from isolated brain slices, spinal cord, small diameter dorsal root ganglion neurons, and injured peripheral nerve neuroma, and furthermore, in the presence of the KCNQ channel blockers, linopirdine or XE-991, this response was blocked [23,25-27]. Moreover, retigabine was shown to improve formalin- or carrageenan-induced pain in rats, which was completely reversed by systemic XE-991 administration [23,28]. These reports suggest that KCNQ channel opening activity may produce an analgesic effect by blocking the action potential throughout the CNS and peripheral nerves. However, the site of action of KCNQ channel openers is not fully clarified thus far. In this study, i.c.v. injection of XE-991 did not affect the analgesic effect of retigabine, despite the same dose preventing retigabine-mediated inhibition of electro convulsion, suppression of exploratory behavior, and rotarod performance. These results suggest that KCNQ channel opening in the brain may play a negligible role in the development of retigabine-induced analgesia in inflammatory pain. Furthermore, we demonstrated that intraplantar administration of retigabine or ICA-27243 suppresses formalin-induced licking behavior. Whereas the contribution of KCNQ channels in the spinal cord cannot be ruled out, these findings suggest opening of KCNQ channels in only the peripheral nerves produces sufficient analgesia. In addition, it’s reported that intraplantar injection of retigabine attenuated bradykinin induced nociceptive behavior [38,39] and that topical injection of the KCNQ channel opener, flupirtine, into the sciatic nerve significantly reversed thermal hyperalgesia in a rat neuropathic pain model induced by partial sciatic nerve ligation [42]. These reports suggest that peripheral KCNQ channels contribute to pain pathway. Moreover, retigabine has been reported to reduce sensitivity to noxious heat of nociceptive A-delta fibers using isolated skin-nerve preparation [43] and the excitability of peripheral human C-fibers [44]. Furthermore, it’s reported that intraplantar injection of XE-991 induced nociceptive behavior and increased the responses of A-delta fibers to noxious heat in the electrophysiological study [38,43,45]. These reports support our hypothesis that KCNQ channel opening within the peripheral nerves controls hyperalgesia. In contrast, Xu et al. [46] reported that i.c.v. injection of retigabine alleviated CFA-induced inflammatory pain in the temporomandibular joint, suggesting the involvement of brain KCNQ channels in retigabine-mediated analgesia. The discrepancies between their and our pain models are unclear. The study of Xu et al. evaluated mechanical allodynia using von Frey filaments whereas our study evaluated thermal hyperalgesia. The difference in pain modality may explain the discrepancy because thermal hyperalgesia during inflammation is considered to result from up-regulation and/or sensitization of the heat-activated channels, transient receptor potential channel, subfamily vanilloid (TRPV) member 1 (TRPV1) and TRPV2, in peripheral nerves [47,48].

Intraplantar injection of formalin in rats is also known to induce two distinct phases of discharges from spinal dorsal horn neurons, which modulate nociceptive behavior and are inhibited by sciatic nerve block [49]. In the CFA-induced inflammatory pain model, inflammatory mediators directly and/or indirectly activate non-selective cation channels (such as TRPV1 and transient receptor potential channel, subfamily A member 1) in Aδ fiber and/or C fibers of primary sensory nerves and thus depolarize neurons [50-52]. The depolarization induces action potentials and causes pain. These findings suggest that both formalin-induced nociceptive responses and CFA-induced pain behavior are caused by action potentials of primary sensory nerves. Retigabine is reported to hyperpolarize the resting membrane potential of primary sensory neurons [23]. Therefore, retigabine is considered to control membrane depolarization and inhibit excessive discharges from inflamed peripheral nerves, thus exhibiting its analgesic effect. This idea is consistent with our present results that intraplantar administration of retigabine inhibited formalin-induced nociceptive responses and i.c.v. injection of XE-991 did not affect the analgesic effect of retigabine on CFA-induced pain.

Retigabine activates all members of the KCNQ channel family except KCNQ1 [12]. ICA-27243 shows more selective activity than retigabine for the KCNQ2/3 subtype by binding to a different site on the KCNQ channel [36,37,53,54]. Roeloffs et al. [37] reported that the anticonvulsant efficacy of ICA-27243 in rodent models was at doses significantly less than those shown to affect open-field locomotor activity or the ability to remain on the rotarod. However, in the present study, ICA-27243 produced an analgesic effect as well as reduced exploratory behavior and impaired motor coordination at the same dose range. Our findings suggest that a selectivity of a KCNQ channel opener toward KCNQ2/3 may be insufficient to separate its analgesic effect with its potential CNS side effects.

In the current study, XE-991 significantly, but only partially, reversed retigabine-induced reduction of exploratory behavior compared with that of motor coordination. The reason for the difference between the two responses is unknown. However, retigabine has been reported to allosterically enhance gamma-aminobutyric acid (GABA) activity for GABA subtype A (GABA_A_) receptors [55-57]. The GABA_A_ agonist, diazepam, was also reported to reduce locomotor activity [58]. Therefore, enhancement of GABA_A_ activity by retigabine may partially contribute to the reduction of exploratory behavior, thus possibly explaining our observation with XE-991 in the present study.

## Conclusion

This study is the first to suggest that peripheral sensory neurons may be the main target for KCNQ channel openers to induce analgesia. Therefore, development of peripheral KCNQ channel openers that do not penetrate the CNS may provide novel analgesic drugs without CNS side effects. While in principal this may be possible with some future KCNQ channel openers, it is unlikely to work for retigabine analogs since retigabine has to penetrate the cell membrane in order to access its binding site on the KCNQ channel [59].

## Methods

### Animals

Adult (6 weeks old) male Sprague–Dawley rats (180–220 g) were purchased from Nihon Clea (Yokohama, Japan) and used according to experimental protocols approved by Takeda’s Experimental Animal Care and Use Committee. The animals were housed in group cages (five rats per cage) under a 12:12 h light/dark cycle (room temperature between 21 and 24.8°C). Water and laboratory chow (type MF; Oriental Yeast Co., Tokyo, Japan) were available ad libitum.

### Surgery for i.c.v. injection

Rats were anesthetized with pentobarbital (50 mg/kg, intraperitoneally) and placed in a stereotaxic apparatus. For i.c.v. cannulation, a small hole was made in the skull with a dental drill and a stainless steel guide cannula (AG-4; Eicom, Kyoto, Japan) was implanted according to stereotaxic coordinates as follows: anterior-posterior = −0.8 mm from bregma, medial-lateral = 1.6 mm right from lambda and dorsal-ventral = 3.8 mm from the skull surface. The guide cannula was set in the skull, and secured with unifast III (03-G0110; GC Corporation). After surgery, the animals were set with a dummy-cannula (AD-4; Eicom) and cap-nut (AC-1; Eicom), and bred (approximately 1 week) in the individual-cage until the day of the experiment. For i.c.v. injection of drugs, the stereo adapter (SAG-10; Eicom) was connected to the Hamilton syringe (80601) through a microinjection tube (JT-10; Eicom), which was inserted in the guide cannula.

### MES test

An electrical stimulus (via ear clip electrodes with a shock level of 95 mA and 100 Hz, and duration of 2 sec) was applied by the ECT unit generator (model 7801 Ugo Basile, Varese, Italy). The stimulation was delivered 30 min after the oral administration of retigabine because the peak of the effect was observed at that time (data not shown). The maximal tonic extension of the hind limbs (tonic extensor convulsions) was taken as an endpoint. If the tonic extensor convulsions did not occur within 5 sec, the animal was regarded to be protected. In control groups (i.e. vehicle treatment) all animals exhibited tonic extension of hind limbs. Therefore, percent inhibition of tonic extensor convulsion was determined by the percentage of the number of the protected animal to the total number.

### Measurement of PWL to thermal stimuli

Rats were placed individually in a clear plastic box on an elevated floor of heat-tempered clear glass (Plantar test, Ugo basile, Varese, Italy). After 30 min of habituation, infrared radiant heat source was focused on the plantar surface of right hind paw. The time of foot withdrawal from the beam of light was measured. The cut-off time in the absence of a response was 25 sec to avoid tissue damage by heating.

### CFA-induced inflammatory pain model

Before CFA injection (−18 hr), baseline PWL was measured, and rats except that with short (< 15 sec) or long (>25 sec) PWL were subcutaneously injected with 200 μL CFA (Sigma-Aldrich, St.Louis, MO) in the plantar surface of the right hind paw using a syringe and a 25-gauge needle. Before administration of compounds (18 h after CFA injection; 0 min), rats were assessed for thermal hyperalgesia, and those with short PWL (< 10 sec) were exposed to the compounds.

### Assessment of exploratory behavior

Locomotor activity was automatically measured using Supermex (Muromachi Kikai, Tokyo, Japan). Rats were administered with either vehicle or the compounds and then temporarily returned to their home cages. To measure their exploratory behavior in a novel environment, the animals were not habituated to the testing cages prior to the experiment. Thirty minutes after oral administration of the compounds, rats were individually placed in test cages for 10 min, and locomotor activities were automatically measured using Supermex.

### Rotarod test

The rotarod test was performed on a rotary apparatus that consisted of a rod (4.3 cm in diameter) suspended horizontally 50 cm above a plane working area. Circular acrylic plate separators were placed at 20-cm intervals along the rod. The rod turned at 18 revolutions per min. Rats were pre-trained 2 days before the experiments, and those that only remained on the rod for 60 sec in at least one of three trials were used in the experiments. The trials (1-min duration) were performed three times and the average time that the animal remained on the rod (≤ 60 sec) was calculated.

### Formalin test

Retigabine and ICA-27243 were dissolved in saline (10% v/v), containing ethanol (20% v/v), Tween 80 (70% v/v). Drug solution or corresponding vehicle (50 μL) was subcutaneously injected in the plantar surface of the right hind paw. Ten minutes after drug administration, the animals then received a subcutaneous injection of 2.5% formalin (50 μL) in the plantar surface of the ipsilateral or contralateral hind paw. A biphasic licking of the injected paw was observed, and the duration of licking was recorded 0–10 min (1st phase) and 10–30 min post-formalin injection (2nd phase).

### Drugs

Retigabine and ICA-27243 (synthesized in the Medicinal Chemistry Research Laboratories of Takeda Pharmaceutical Company, Osaka, Japan) were formulated in methylcellulose, and administered orally (5 mL/kg) 30 min prior to the behavioral test, unless otherwise stated. Retigabine was orally administrated 50 mg/kg in XE-991 combination rotarod study and 20 mg/kg in another combination study. The selective K_v_7 blocker was 10, 10-*bis* (4-Pyridinylmethyl)-9(10*H*)-anthracenone dihydrochloride (XE-991) (TOCRIS bioscience, Bristol, UK). XE-991 was dissolved in saline, and administered (i.c.v.) (20 μL total volume, infusion rate of 40 μL/min) 20 min prior to the behavioral test.

### Statistical analysis

Data are expressed as the mean ± S.E.M. Between-group differences were analyzed with the Williams’ test, Shirley-Williams test, Dunnett’s test, non-parametric Dunnett’s test, Student’s t-test or Welch’s t-test. Significance was reached at values of *p* < 0.05 and *p* < 0.01.

## Abbreviations

CNS: Central nervous system; i.c.v.: Intracerebroventricular; CFA: Complete Freund’s adjuvant; PWL: Paw withdrawal latency; MES: Maximal electroshock seizure.

## Competing interests

The authors declare that they have no competing interests.

## Authors’ contributions

HH designed and contributed all experiments and performed MES test, locomotor activity test and plantar test and analysed these experiments data. MI designed and performed rota rod test and formalin test and analysed these experiments data. TM contributed to all experiments design and supervised research. HH wrote the manuscript. Above all authors and NT contributed read and approved the final manuscript.
